# Poly[[[μ-1,4-bis­(pyridin-4-ylcarbon­yl)piperazine-κ^2^ *N*:*N*′][μ-2-(2-carboxyl­atoeth-1-en-1-yl)benzoato-κ^2^ *O*:*O* ^2^]zinc(II)] 2.5 hydrate]: a tri-periodic coordination polymer with a dimer-based six-connected pcu topology

**DOI:** 10.1107/S2414314623008118

**Published:** 2023-09-22

**Authors:** Erika J. Glatz, Robert L. LaDuca

**Affiliations:** a E-35 Holmes Hall, Michigan State University, Lyman Briggs College, 919 E. Shaw Lane, East Lansing, MI 48825, USA; Sunway University, Malaysia

**Keywords:** crystal structure, tri-periodic coordination polymer, **pcu** topology, cca, 4-pcap

## Abstract

The title compound, {[Zn(C_10_H_6_O_4_)(C_16_H_16_N_4_O_2_]·2.5H_2_O}_*n*_, contains five-coordinate Zn^II^ ions inter­mediate between square-pyramidal and trigonal–bipyramidal coordination environments. The Zn^II^ ions are connected by 2-carb­oxy­cinnamate (cca) ligands and *N*,*N*′-bis-(pyridine-4-carboxamido)­piperazine (4-pcap) ligands to construct a non-inter­penetrated, tri-periodic coordination polymer with embedded [Zn_2_(OCO)_2_] dimeric units. Treating these as six-connected nodes reveals an overall (4^12^6^3^) **pcu** topology. One of the 4-pcap piperazinyl rings is disordered equally over two sets of crystallographic positions.

## Abstract 2

The title compound contains five-coordinate Zn^II^ ions inter­mediate between square-pyramidal and trigonal–bipyramidal coordination geometries. The Zn^II^ ions are connected by 2-carb­oxy­cinnamate (cca) ligands and *N*,*N*′-bis-(pyridine-4-carboxamido)­piperazine (4-pcap) ligands to construct a non-inter­penetrated, tri-periodic coordination polymer with embedded [Zn_2_(OCO)_2_] dimeric units. Treating these as six-connected nodes reveals an overall (4^12^6^3^) **pcu** topology.

## Graphical abstract

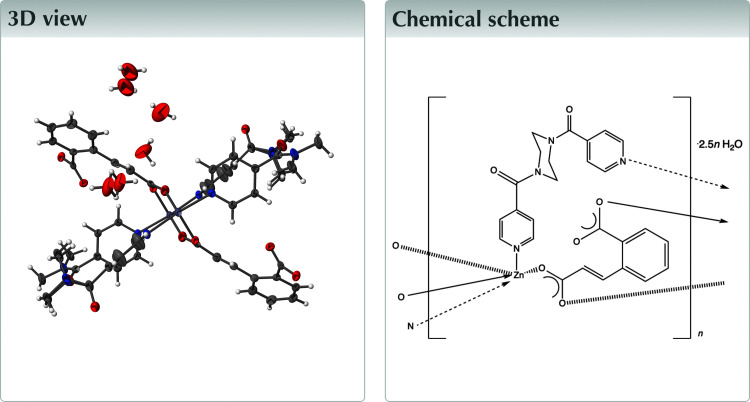

## Structure description

Previously our group reported a series of coordination polymers synthesized from 2-carb­oxy­cinnamic acid (ccaH_2_) and *N*-(pyridin-3-yl)isonicotinamide (3-pina), which resulted in *in situ* lactonization of the ccaH_2_ to form 1,3-di­hydro-3-oxo-1-isobenzo­furan­acetate (dibf). The final crystallized products contained di-periodic layered coordination polymers of formulation [*M*(dibf)_2_(3-pina)_2_]_*n*_, where *M* = Zn, Cd, Mn, Co, and Ni (Murray & LaDuca, 2014 ▸). The title complex was obtained during attempts to prepare a divalent zinc coordination polymer containing cca and *N*,*N*′-bis-(pyridine-4-carboxamido)­piperazine (4-pcap) ligands. Isomerization of ccaH_2_ to dibf did not occur during the synthesis of the title compound.

**Figure 1:**
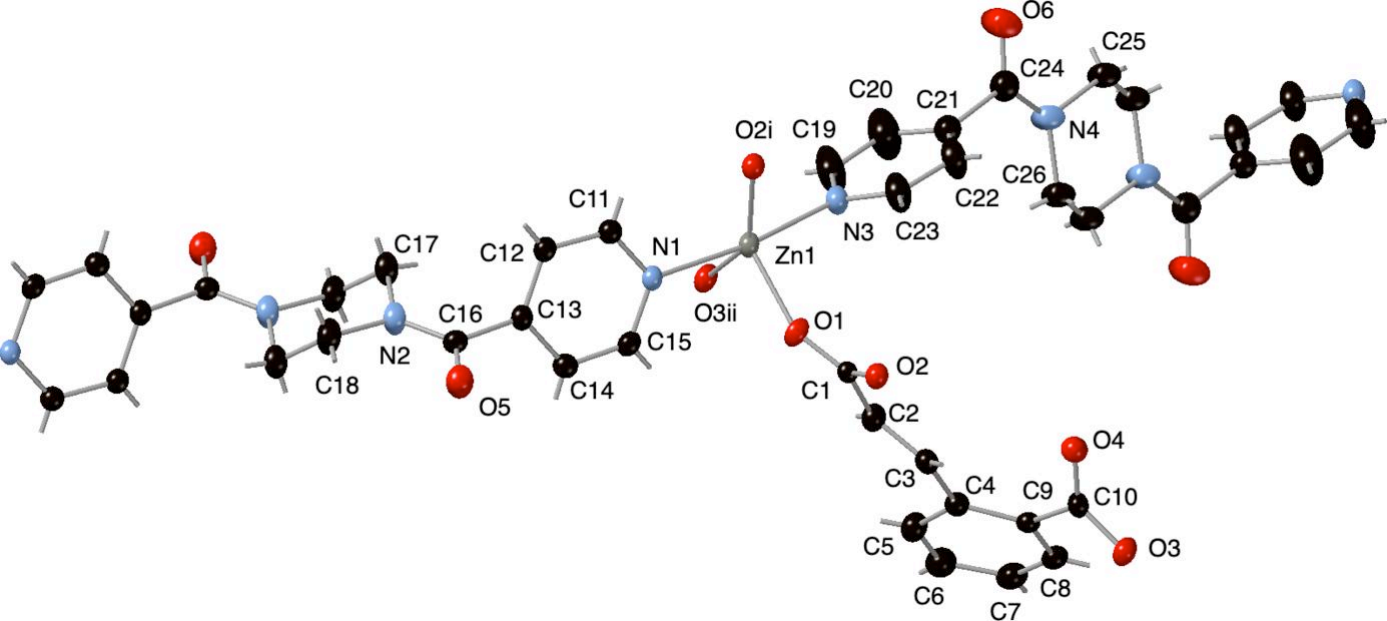
Zinc coordination environment in the title compound with full ligand set. Displacement ellipsoids are drawn at the 50% probability level. Only one disordered component of a 4-pcap ligand’s piperazinyl ring (N4, C25, C26) is shown. Color code: Zn, gray; O, red; N, light blue; C, black. H-atom positions are shown as gray sticks. Symmetry codes are as listed in Table 1 ▸.

The title compound displays an asymmetric unit containing a five-coordinate Zn^II^ ion, a complete cca ligand, and halves of two crystallographically distinct 4-pcap ligands whose chair-conformation piperazinyl rings are situated about crystallographic inversion centers (Fig. 1 ▸). In one of these 4-pcap ligands, the piperazinyl ring atoms (N4, C25, C26) are disordered equally over two sets of positions. The Zn^II^ ion is five-coordinate inter­mediate between square-pyramidal and trigonal–bipyramidal, with a τ trigonality index of 0.45 (Addison *et al.*, 1984 ▸). Two of the ‘trans’ coordination sites are occupied by pyridyl N-atom donors belonging to crystallographically distinct 4-pcap ligands. The other three coordination sites are taken up by carboxyl­ate O-atom donors belonging to three distinct cca ligands. Bond lengths and angles within the coordination environment at Zn are listed in Table 1 ▸. Bridging carboxyl­ate groups from cca ligands form [Zn_2_(OCO)_2_] dimeric units with a Zn⋯Zn through-space distance of 4.360 (1) Å.

**Figure 2:**
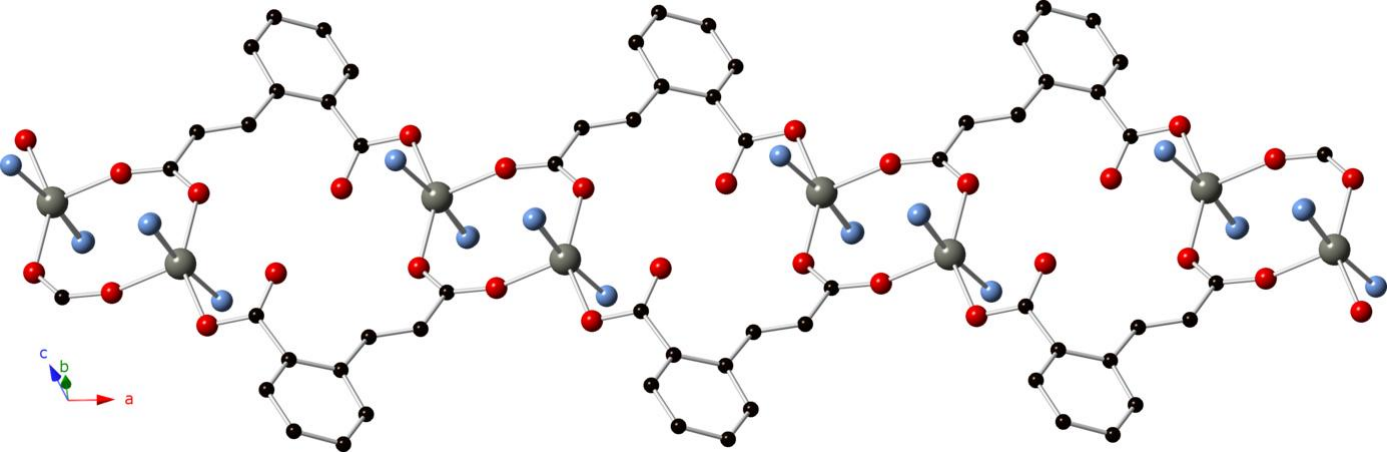
[Zn_2_(cca)_2_]_*n*_ mono-periodic coordination polymer chain motif in the title compound.

**Figure 3:**
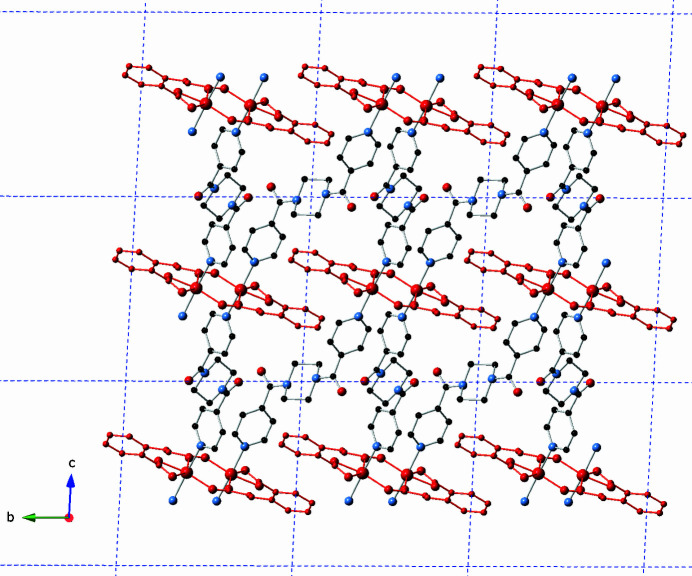
[Zn(cca)(4-pcap)]_*n*_ tri-periodic coordination polymer network in the title compound. The [Zn_2_(cca)_2_]_*n*_ chain motifs are shown in red.

**Figure 4:**
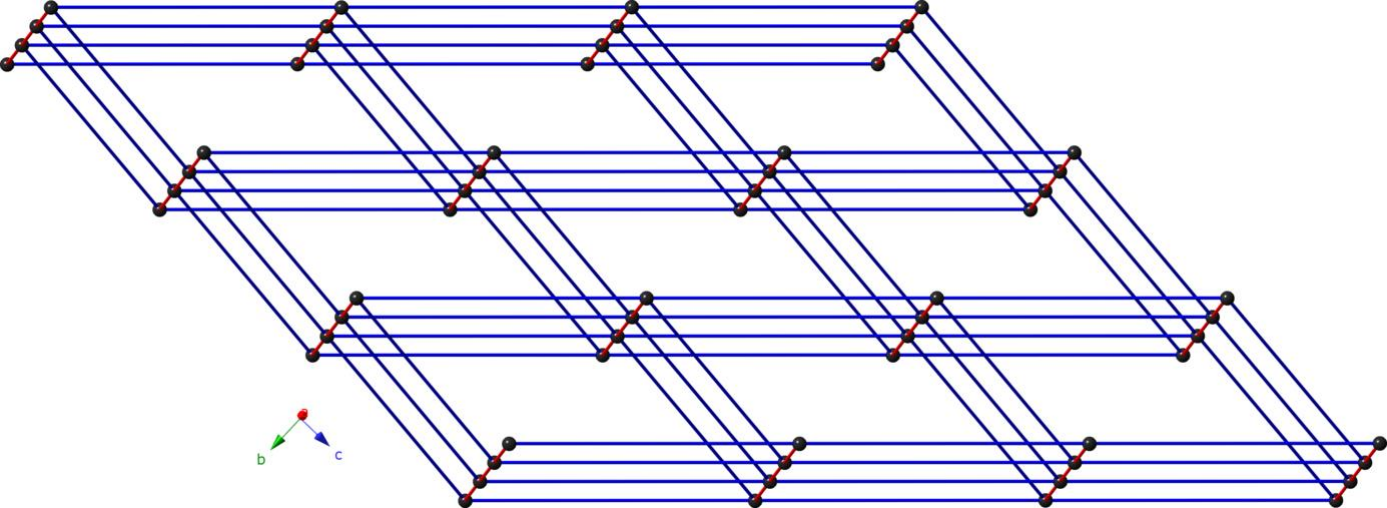
Schematic perspective of the 6-connected **pcu** topology in the title compound.

The full span of the cca ligands connect these dimeric units into [Zn_2_(cca)_2_]_*n*_ mono-periodic coordination polymer chains oriented along the *a* axis (Fig. 2 ▸). The chain motifs are linked into a tri-periodic non-inter­penetrated coordination polymer network with formulation [Zn(cca)(4-pcap)]_*n*_ by the 4-pcap ligands (Fig. 3 ▸). Water mol­ecules of crystallization with partial occupancy are anchored to the coordination polymer network by donating hydrogen bonds to cca carboxyl­ate O atoms and 4-pcap carboxamide O atoms (Table 2 ▸). Considering the [Zn_2_(OCO)_2_] dimeric units as 6-connected nodes results in a (4^12^6^3^) **pcu** topology for the title compound, as determined by inspection (Fig. 4 ▸).

## Synthesis and crystallization

Zn(NO_3_)_2_^.^6H_2_O (110 mg, 0.37 mmol), 2-carb­oxy­cinnamic acid (ccaH_2_) (71 mg, 0.37 mmol), *N*,*N*′-bis-(pyridine-4-carbox­amido)­piperazine (4-pcap) (110 mg, 0.37 mmol), and 0.75 ml of a 1.0 *M* NaOH solution were placed into 10 ml of distilled water in a Teflon-lined acid digestion bomb. The bomb was sealed and heated in an oven at 393 K for 48 h, and then cooled slowly to 273 K. Colorless crystals of the title complex were obtained in 54% yield.

## Refinement

Crystal data, data collection and structure refinement details are summarized in Table 3 ▸. All H atoms attached to C atoms were placed in calculated positions and refined with a riding model. The H atoms belonging to water mol­ecules of crystallization O1*W* and O2*W* were placed in calculated positions and refined with a riding model. The H atoms belonging to water mol­ecules of crystallization O3*W* and O4*W* were placed in calculated positions and then refined with fixed positions. The piperazinyl ring in one of the 4-pcap ligands was disordered equally over two sets of positions and was treated using PART commands. EADP commands were used to enforce identical atomic displacement parameters for the C and N atoms involved in the disorder, in order to avoid non-positive definite *U*_ij_ values.

## Supplementary Material

Crystal structure: contains datablock(s) I, 1R. DOI: 10.1107/S2414314623008118/tk4097sup1.cif

Structure factors: contains datablock(s) I. DOI: 10.1107/S2414314623008118/tk4097Isup2.hkl

CCDC reference: 1492691

Additional supporting information:  crystallographic information; 3D view; checkCIF report

## Figures and Tables

**Table 1:** Selected geometric parameters (Å, °)

Zn1—O1	2.020 (2)	Zn1—N1	2.169 (2)
Zn1—O2^i^	2.0249 (19)	Zn1—N3	2.139 (2)
Zn1—O3^ii^	2.0555 (19)		
			
O1—Zn1—O2^i^	112.24 (8)	O2^i^—Zn1—N1	87.90 (8)
O1—Zn1—O3^ii^	101.58 (8)	O2^i^—Zn1—N3	89.27 (8)
O1—Zn1—N1	90.05 (8)	O3^ii^—Zn1—N1	86.39 (8)
O1—Zn1—N3	97.32 (9)	O3^ii^—Zn1—N3	92.14 (8)
O2^i^—Zn1—O3^ii^	145.68 (8)	N3—Zn1—N1	172.63 (8)

Symmetry codes: (i) 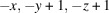 ; (ii) 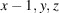 .

**Table 2:** Hydrogen-bond geometry (Å, °)

*D*—H⋯*A*	*D*—H	H⋯*A*	*D*⋯*A*	*D*—H⋯*A*
O2*W*—H2*WA*⋯O3^ii^	0.87	1.87	2.732 (4)	172
O2*W*—H2*WB*⋯O3*W*	0.87	1.92	2.770 (7)	165
O3*W*—H3*WA*⋯O4*W*	0.87	1.85	2.585 (13)	140
O3*W*—H3*WB*⋯O5^iii^	0.87	2.08	2.888 (5)	154

Symmetry codes: (ii) 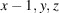 ; (iii) 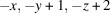 .

**Table 3:** Experimental details

Crystal data	
Chemical formula	[Zn(C_10_H_6_O_4_)(C_16_H_16_N_4_O_2_]·2.5H_2_O
*M*_r_	596.88
Crystal system, space group	Triclinic, *P* 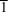
Temperature (K)	173
*a*, *b*, *c* (Å)	9.7753 (7), 11.2349 (8), 12.6422 (9)
α, β, γ (°)	90.946 (1), 111.500 (1), 94.586 (1)
*V* (Å^3^)	1286.11 (16)
*Z*	2
Radiation type	Mo *K*α
μ (mm^−1^)	1.02
Crystal size (mm)	0.32 × 0.18 × 0.12

Data collection	
Diffractometer	Bruker APEXII CCD
Absorption correction	Multi-scan (*SADABS*; Krause *et al.*, 2015 ▸)
*T*_min_, *T*_max_	0.683, 0.745
No. of measured, independent and observed [*I* > 2σ(*I*)] reflections	21745, 4731, 4072
*R*_int_	0.042
(sin θ/λ)_max_ (Å^−1^)	0.602

Refinement	
*R*[*F* ^2^ > 2σ(*F* ^2^)], *wR*(*F* ^2^), *S*	0.042, 0.109, 1.05
No. of reflections	4731
No. of parameters	379
H-atom treatment	H atoms treated by a mixture of independent and constrained refinement
Δρ_max_, Δρ_min_ (e Å^−3^)	0.59, −0.44

Computer programs: *APEX2* and *SAINT* (Bruker, 2014 ▸), *SHELXT* (Sheldrick, 2015*a* ▸), *SHELXL* (Sheldrick, 2015*b* ▸), *CrystalMaker X* (Palmer, 2020 ▸), and *OLEX2* (Dolomanov *et al.*, 2009 ▸).

## full crystallographic data

### Crystal data

**Table:**

[Zn(C_10_H_6_O_4_)(C_16_H_16_N_4_O_2_]·2.5H_2_O	*Z* = 2
*M**_r_* = 596.88	*F*(000) = 618
Triclinic, *P*1	*D*_x_ = 1.541 Mg m^−^^3^
*a* = 9.7753 (7) Å	Mo *K*α radiation, λ = 0.71073 Å
*b* = 11.2349 (8) Å	Cell parameters from 9996 reflections
*c* = 12.6422 (9) Å	θ = 2.3–25.3°
α = 90.946 (1)°	µ = 1.02 mm^−^^1^
β = 111.500 (1)°	*T* = 173 K
γ = 94.586 (1)°	Plate, colourless
*V* = 1286.11 (16) Å^3^	0.32 × 0.18 × 0.12 mm

### Data collection

**Table:**

Bruker APEXII CCD diffractometer	4072 reflections with *I* > 2σ(*I*)
φ and ω scans	*R*_int_ = 0.042
Absorption correction: multi-scan (SADABS; Krause *et al.*, 2015)	θ_max_ = 25.4°, θ_min_ = 1.7°
*T*_min_ = 0.683, *T*_max_ = 0.745	*h* = −11→11
21745 measured reflections	*k* = −13→13
4731 independent reflections	*l* = −15→15

### Refinement

**Table:**

Refinement on *F*^2^	0 restraints
Least-squares matrix: full	Hydrogen site location: mixed
*R*[*F*^2^ > 2σ(*F*^2^)] = 0.042	H atoms treated by a mixture of independent and constrained refinement
*wR*(*F*^2^) = 0.109	*w* = 1/[σ^2^(*F*_o_^2^) + (0.0576*P*)^2^ + 1.1933*P*] where *P* = (*F*_o_^2^ + 2*F*_c_^2^)/3
*S* = 1.05	(Δ/σ)_max_ = 0.001
4731 reflections	Δρ_max_ = 0.59 e Å^−^^3^
379 parameters	Δρ_min_ = −0.44 e Å^−^^3^

### Special details

**Table:**

Geometry. All esds (except the esd in the dihedral angle between two l.s. planes) are estimated using the full covariance matrix. The cell esds are taken into account individually in the estimation of esds in distances, angles and torsion angles; correlations between esds in cell parameters are only used when they are defined by crystal symmetry. An approximate (isotropic) treatment of cell esds is used for estimating esds involving l.s. planes.

### Fractional atomic coordinates and isotropic or equivalent isotropic displacement parameters (Å^2^)

**Table:**

	*x*	*y*	*z*	*U*_iso_*/*U*_eq_	Occ. (<1)
Zn1	−0.15546 (3)	0.62686 (3)	0.50462 (3)	0.02129 (12)	
O1	0.0637 (2)	0.6167 (2)	0.58481 (18)	0.0335 (5)	
O2	0.2592 (2)	0.52624 (16)	0.58582 (16)	0.0243 (4)	
O3	0.8181 (2)	0.79023 (17)	0.56657 (17)	0.0267 (4)	
O4	0.5903 (2)	0.70545 (17)	0.48281 (17)	0.0280 (4)	
O5	−0.1940 (2)	0.3588 (2)	0.99632 (19)	0.0423 (6)	
O6	−0.2907 (3)	0.7848 (3)	−0.0556 (2)	0.0754 (10)	
N1	−0.1944 (2)	0.5527 (2)	0.64955 (19)	0.0214 (5)	
N2	−0.4061 (3)	0.4473 (2)	0.9549 (2)	0.0343 (6)	
N3	−0.1472 (3)	0.7006 (2)	0.3520 (2)	0.0243 (5)	
N4	−0.0913 (12)	0.9139 (10)	0.0150 (10)	0.055 (2)	0.5
N4A	−0.0617 (12)	0.8707 (10)	0.0061 (10)	0.055 (2)	0.5
C1	0.1968 (3)	0.6189 (3)	0.5937 (2)	0.0229 (6)	
C2	0.2851 (3)	0.7357 (3)	0.6161 (2)	0.0269 (6)	
H2	0.238736	0.804934	0.623152	0.032*	
C3	0.4259 (3)	0.7495 (2)	0.6269 (2)	0.0230 (6)	
H3	0.473237	0.679361	0.624748	0.028*	
C4	0.5140 (3)	0.8655 (2)	0.6420 (2)	0.0230 (6)	
C5	0.4832 (3)	0.9620 (3)	0.6986 (3)	0.0316 (7)	
H5	0.410901	0.949627	0.731975	0.038*	
C6	0.5551 (3)	1.0745 (3)	0.7070 (3)	0.0340 (7)	
H6	0.532354	1.138200	0.746211	0.041*	
C7	0.6603 (3)	1.0948 (3)	0.6585 (3)	0.0301 (7)	
H7	0.705651	1.173136	0.660143	0.036*	
C8	0.6988 (3)	1.0000 (2)	0.6077 (2)	0.0247 (6)	
H8	0.774332	1.013093	0.577604	0.030*	
C9	0.6284 (3)	0.8850 (2)	0.5999 (2)	0.0199 (5)	
C10	0.6782 (3)	0.7860 (2)	0.5446 (2)	0.0217 (6)	
C11	−0.3122 (3)	0.4749 (3)	0.6345 (2)	0.0256 (6)	
H11	−0.372659	0.446765	0.559249	0.031*	
C12	−0.3492 (3)	0.4339 (3)	0.7242 (2)	0.0274 (6)	
H12	−0.434016	0.379278	0.710359	0.033*	
C13	−0.2611 (3)	0.4735 (3)	0.8342 (2)	0.0247 (6)	
C14	−0.1389 (3)	0.5547 (3)	0.8502 (2)	0.0285 (6)	
H14	−0.076211	0.584059	0.924517	0.034*	
C15	−0.1109 (3)	0.5916 (3)	0.7564 (2)	0.0258 (6)	
H15	−0.027963	0.647579	0.767864	0.031*	
C16	−0.2858 (3)	0.4230 (3)	0.9354 (2)	0.0280 (6)	
C17	−0.5221 (4)	0.5169 (3)	0.8825 (3)	0.0398 (8)	
H17A	−0.611224	0.462603	0.840273	0.048*	
H17B	−0.488164	0.557330	0.826334	0.048*	
C18	−0.4399 (4)	0.3913 (3)	1.0475 (3)	0.0393 (8)	
H18A	−0.353374	0.351967	1.096714	0.047*	
H18B	−0.523926	0.329325	1.015340	0.047*	
C19	−0.2282 (5)	0.7892 (3)	0.3032 (3)	0.0560 (11)	
H19	−0.281012	0.826011	0.342737	0.067*	
C20	−0.2390 (5)	0.8297 (4)	0.1985 (4)	0.0632 (12)	
H20	−0.300901	0.891042	0.166220	0.076*	
C21	−0.1611 (3)	0.7821 (3)	0.1415 (3)	0.0333 (7)	
C22	−0.0800 (4)	0.6893 (3)	0.1895 (3)	0.0476 (9)	
H22	−0.026352	0.651547	0.151260	0.057*	
C23	−0.0767 (4)	0.6512 (3)	0.2932 (3)	0.0422 (9)	
H23	−0.021078	0.586034	0.324248	0.051*	
C24	−0.1805 (4)	0.8208 (3)	0.0238 (3)	0.0427 (8)	
C25	−0.1099 (11)	0.9534 (11)	−0.1007 (9)	0.055 (2)	0.5
H25A	−0.076411	0.891093	−0.139522	0.066*	0.5
H25B	−0.216754	0.957040	−0.143984	0.066*	0.5
C25A	0.0797 (12)	0.9105 (10)	0.0941 (10)	0.055 (2)	0.5
H25C	0.162575	0.890943	0.071708	0.066*	0.5
H25D	0.090287	0.874146	0.167210	0.066*	0.5
C26	0.0516 (12)	0.9594 (10)	0.1064 (10)	0.055 (2)	0.5
H26C	0.033841	0.962963	0.178519	0.066*	0.5
H26D	0.121296	0.897875	0.113890	0.066*	0.5
H26A	−0.181 (11)	0.894 (8)	−0.158 (8)	0.082*	0.5
H26B	0.016 (11)	0.886 (8)	−0.124 (8)	0.082*	0.5
C26A	−0.0701 (11)	0.9102 (12)	−0.1049 (9)	0.055 (2)	0.5
O1W	0.0231 (8)	0.9573 (7)	0.5214 (8)	0.084 (2)	0.5
H1WA	−0.029169	0.917347	0.553973	0.126*	0.5
H1WB	−0.035607	0.958220	0.450405	0.126*	0.5
O2W	0.0639 (5)	0.9328 (4)	0.6978 (4)	0.0783 (13)	0.75
H2WA	−0.018142	0.889470	0.660137	0.117*	0.75
H2WB	0.110935	0.899043	0.760734	0.117*	0.75
O3W	0.2431 (5)	0.8664 (4)	0.9103 (5)	0.1026 (18)	0.75
H3WA	0.338476	0.865930	0.933176	0.154*	0.75
H3WB	0.211426	0.792850	0.915446	0.154*	0.75
O4W	0.5073 (13)	0.9704 (11)	0.9673 (7)	0.117 (4)	0.5
H4WA	0.569979	0.996946	0.937002	0.176*	0.5
H4WB	0.520409	0.894976	0.975842	0.176*	0.5

### Atomic displacement parameters (Å^2^)

**Table:**

	*U*^11^	*U*^22^	*U*^33^	*U*^12^	*U*^13^	*U*^23^
Zn1	0.02256 (19)	0.02113 (19)	0.02374 (19)	0.00199 (12)	0.01265 (14)	0.00330 (13)
O1	0.0190 (10)	0.0462 (13)	0.0380 (12)	0.0021 (9)	0.0135 (9)	0.0066 (10)
O2	0.0244 (10)	0.0236 (10)	0.0272 (10)	0.0003 (8)	0.0128 (8)	0.0004 (8)
O3	0.0231 (10)	0.0238 (10)	0.0375 (12)	0.0020 (8)	0.0162 (9)	0.0005 (9)
O4	0.0299 (11)	0.0253 (11)	0.0282 (11)	−0.0009 (9)	0.0110 (9)	−0.0042 (9)
O5	0.0352 (12)	0.0607 (16)	0.0360 (13)	0.0133 (11)	0.0166 (10)	0.0184 (11)
O6	0.073 (2)	0.101 (2)	0.0319 (14)	−0.0514 (18)	0.0073 (14)	0.0112 (15)
N1	0.0218 (11)	0.0226 (12)	0.0232 (12)	0.0028 (9)	0.0119 (10)	0.0020 (9)
N2	0.0335 (14)	0.0461 (16)	0.0317 (14)	0.0104 (12)	0.0199 (12)	0.0160 (12)
N3	0.0303 (13)	0.0205 (12)	0.0272 (13)	0.0037 (10)	0.0162 (10)	0.0050 (10)
N4	0.036 (3)	0.090 (6)	0.0246 (16)	−0.021 (3)	−0.0004 (19)	0.025 (3)
N4A	0.036 (3)	0.090 (6)	0.0246 (16)	−0.021 (3)	−0.0004 (19)	0.025 (3)
C1	0.0194 (14)	0.0339 (16)	0.0157 (13)	0.0009 (12)	0.0071 (11)	0.0042 (11)
C2	0.0269 (15)	0.0257 (15)	0.0319 (16)	0.0049 (12)	0.0147 (13)	−0.0005 (12)
C3	0.0263 (14)	0.0221 (14)	0.0220 (14)	0.0043 (11)	0.0102 (12)	0.0011 (11)
C4	0.0228 (14)	0.0224 (14)	0.0243 (14)	0.0054 (11)	0.0084 (12)	0.0008 (11)
C5	0.0296 (16)	0.0292 (16)	0.0389 (18)	0.0022 (13)	0.0166 (14)	−0.0028 (13)
C6	0.0348 (17)	0.0253 (15)	0.0418 (18)	0.0049 (13)	0.0142 (14)	−0.0075 (13)
C7	0.0297 (16)	0.0201 (14)	0.0366 (17)	−0.0007 (12)	0.0080 (13)	0.0012 (12)
C8	0.0213 (14)	0.0240 (14)	0.0264 (15)	0.0013 (11)	0.0061 (12)	0.0040 (12)
C9	0.0179 (13)	0.0209 (13)	0.0188 (13)	0.0041 (10)	0.0037 (11)	0.0014 (10)
C10	0.0253 (14)	0.0226 (14)	0.0203 (14)	0.0027 (11)	0.0116 (11)	0.0066 (11)
C11	0.0245 (14)	0.0279 (15)	0.0249 (15)	−0.0005 (12)	0.0102 (12)	−0.0008 (12)
C12	0.0246 (14)	0.0323 (16)	0.0265 (15)	−0.0053 (12)	0.0124 (12)	−0.0017 (12)
C13	0.0235 (14)	0.0291 (15)	0.0244 (15)	0.0033 (12)	0.0120 (12)	0.0014 (12)
C14	0.0262 (15)	0.0334 (16)	0.0248 (15)	−0.0015 (12)	0.0094 (12)	−0.0018 (12)
C15	0.0214 (14)	0.0267 (15)	0.0299 (16)	−0.0029 (11)	0.0114 (12)	−0.0010 (12)
C16	0.0230 (14)	0.0353 (17)	0.0239 (15)	−0.0034 (12)	0.0076 (12)	0.0010 (13)
C17	0.0353 (17)	0.061 (2)	0.0318 (17)	0.0126 (16)	0.0196 (14)	0.0180 (16)
C18	0.0392 (18)	0.047 (2)	0.0425 (19)	0.0087 (15)	0.0259 (16)	0.0181 (16)
C19	0.088 (3)	0.053 (2)	0.053 (2)	0.043 (2)	0.048 (2)	0.0306 (19)
C20	0.095 (3)	0.058 (3)	0.062 (3)	0.044 (2)	0.050 (3)	0.036 (2)
C21	0.0329 (16)	0.0368 (17)	0.0301 (17)	−0.0035 (13)	0.0127 (13)	0.0100 (13)
C22	0.061 (2)	0.058 (2)	0.040 (2)	0.0255 (19)	0.0337 (18)	0.0167 (17)
C23	0.054 (2)	0.048 (2)	0.0395 (19)	0.0288 (17)	0.0295 (17)	0.0206 (16)
C24	0.0389 (19)	0.055 (2)	0.0337 (19)	−0.0049 (16)	0.0143 (16)	0.0162 (16)
C25	0.036 (3)	0.090 (6)	0.0246 (16)	−0.021 (3)	−0.0004 (19)	0.025 (3)
C25A	0.036 (3)	0.090 (6)	0.0246 (16)	−0.021 (3)	−0.0004 (19)	0.025 (3)
C26	0.036 (3)	0.090 (6)	0.0246 (16)	−0.021 (3)	−0.0004 (19)	0.025 (3)
C26A	0.036 (3)	0.090 (6)	0.0246 (16)	−0.021 (3)	−0.0004 (19)	0.025 (3)
O1W	0.069 (5)	0.072 (5)	0.122 (7)	−0.012 (4)	0.050 (5)	0.016 (5)
O2W	0.054 (3)	0.071 (3)	0.093 (4)	−0.011 (2)	0.011 (2)	−0.020 (2)
O3W	0.082 (3)	0.063 (3)	0.136 (5)	0.010 (2)	0.007 (3)	0.031 (3)
O4W	0.109 (6)	0.157 (11)	0.081 (8)	0.009 (7)	0.029 (6)	0.042 (7)

### Geometric parameters (Å, º)

**Table:**

Zn1—O1	2.020 (2)	C11—C12	1.387 (4)
Zn1—O2^i^	2.0249 (19)	C12—H12	0.9500
Zn1—O3^ii^	2.0555 (19)	C12—C13	1.384 (4)
Zn1—N1	2.169 (2)	C13—C14	1.394 (4)
Zn1—N3	2.139 (2)	C13—C16	1.499 (4)
O1—C1	1.262 (3)	C14—H14	0.9500
O2—C1	1.269 (3)	C14—C15	1.375 (4)
O3—C10	1.289 (3)	C15—H15	0.9500
O4—C10	1.236 (3)	C17—H17A	0.9900
O5—C16	1.238 (4)	C17—H17B	0.9900
O6—C24	1.207 (4)	C17—C18^iii^	1.502 (5)
N1—C11	1.341 (4)	C18—H18A	0.9900
N1—C15	1.341 (4)	C18—H18B	0.9900
N2—C16	1.334 (4)	C19—H19	0.9500
N2—C17	1.462 (4)	C19—C20	1.377 (5)
N2—C18	1.466 (4)	C20—H20	0.9500
N3—C19	1.337 (4)	C20—C21	1.357 (5)
N3—C23	1.326 (4)	C21—C22	1.373 (5)
N4—C24	1.340 (13)	C21—C24	1.506 (4)
N4—C25	1.486 (10)	C22—H22	0.9500
N4—C26	1.494 (10)	C22—C23	1.376 (5)
N4A—C24	1.343 (12)	C23—H23	0.9500
N4A—C25A	1.451 (10)	C25—H25A	0.9900
N4A—C26A	1.454 (10)	C25—H25B	0.9900
C1—C2	1.475 (4)	C25—C26^iv^	1.11 (2)
C2—H2	0.9500	C25A—H25C	0.9900
C2—C3	1.328 (4)	C25A—H25D	0.9900
C3—H3	0.9500	C25A—C26A^iv^	2.029 (19)
C3—C4	1.473 (4)	C26—H26C	0.9900
C4—C5	1.400 (4)	C26—H26D	0.9900
C4—C9	1.409 (4)	C26A—H26A	1.05 (10)
C5—H5	0.9500	C26A—H26B	1.01 (9)
C5—C6	1.378 (4)	O1W—H1WA	0.8694
C6—H6	0.9500	O1W—H1WB	0.8705
C6—C7	1.384 (4)	O2W—H2WA	0.8702
C7—H7	0.9500	O2W—H2WB	0.8706
C7—C8	1.380 (4)	O3W—H3WA	0.8699
C8—H8	0.9500	O3W—H3WB	0.8704
C8—C9	1.398 (4)	O4W—H4WA	0.8697
C9—C10	1.508 (4)	O4W—H4WB	0.8700
C11—H11	0.9500		
			
O1—Zn1—O2^i^	112.24 (8)	C15—C14—C13	118.8 (3)
O1—Zn1—O3^ii^	101.58 (8)	C15—C14—H14	120.6
O1—Zn1—N1	90.05 (8)	N1—C15—C14	123.4 (3)
O1—Zn1—N3	97.32 (9)	N1—C15—H15	118.3
O2^i^—Zn1—O3^ii^	145.68 (8)	C14—C15—H15	118.3
O2^i^—Zn1—N1	87.90 (8)	O5—C16—N2	123.0 (3)
O2^i^—Zn1—N3	89.27 (8)	O5—C16—C13	118.1 (3)
O3^ii^—Zn1—N1	86.39 (8)	N2—C16—C13	118.9 (3)
O3^ii^—Zn1—N3	92.14 (8)	N2—C17—H17A	109.5
N3—Zn1—N1	172.63 (8)	N2—C17—H17B	109.5
C1—O1—Zn1	156.27 (19)	N2—C17—C18^iii^	110.9 (3)
C1—O2—Zn1^i^	125.25 (17)	H17A—C17—H17B	108.1
C10—O3—Zn1^v^	103.62 (16)	C18^iii^—C17—H17A	109.5
C11—N1—Zn1	120.68 (18)	C18^iii^—C17—H17B	109.5
C15—N1—Zn1	121.34 (18)	N2—C18—C17^iii^	110.6 (3)
C15—N1—C11	117.6 (2)	N2—C18—H18A	109.5
C16—N2—C17	125.3 (2)	N2—C18—H18B	109.5
C16—N2—C18	120.2 (3)	C17^iii^—C18—H18A	109.5
C17—N2—C18	114.1 (2)	C17^iii^—C18—H18B	109.5
C19—N3—Zn1	121.5 (2)	H18A—C18—H18B	108.1
C23—N3—Zn1	121.7 (2)	N3—C19—H19	118.4
C23—N3—C19	116.3 (3)	N3—C19—C20	123.2 (3)
C24—N4—C25	118.1 (8)	C20—C19—H19	118.4
C24—N4—C26	124.7 (7)	C19—C20—H20	120.0
C25—N4—C26	114.8 (6)	C21—C20—C19	120.0 (3)
C24—N4A—C25A	125.6 (7)	C21—C20—H20	120.0
C24—N4A—C26A	122.0 (7)	C20—C21—C22	117.3 (3)
C25A—N4A—C26A	111.6 (6)	C20—C21—C24	119.9 (3)
O1—C1—O2	123.7 (3)	C22—C21—C24	122.3 (3)
O1—C1—C2	118.1 (3)	C21—C22—H22	120.1
O2—C1—C2	118.2 (2)	C21—C22—C23	119.7 (3)
C1—C2—H2	118.2	C23—C22—H22	120.1
C3—C2—C1	123.7 (3)	N3—C23—C22	123.4 (3)
C3—C2—H2	118.2	N3—C23—H23	118.3
C2—C3—H3	117.6	C22—C23—H23	118.3
C2—C3—C4	124.8 (3)	O6—C24—N4	121.3 (6)
C4—C3—H3	117.6	O6—C24—N4A	120.5 (6)
C5—C4—C3	119.7 (3)	O6—C24—C21	119.4 (3)
C5—C4—C9	117.7 (3)	N4—C24—C21	117.6 (6)
C9—C4—C3	122.6 (2)	N4A—C24—C21	118.3 (6)
C4—C5—H5	119.1	N4—C25—H25A	108.0
C6—C5—C4	121.7 (3)	N4—C25—H25B	108.0
C6—C5—H5	119.1	H25A—C25—H25B	107.2
C5—C6—H6	119.9	C26^iv^—C25—N4	117.2 (12)
C5—C6—C7	120.2 (3)	C26^iv^—C25—H25A	108.0
C7—C6—H6	119.9	C26^iv^—C25—H25B	108.0
C6—C7—H7	120.3	N4A—C25A—H25C	111.3
C8—C7—C6	119.4 (3)	N4A—C25A—H25D	111.3
C8—C7—H7	120.3	N4A—C25A—C26A^iv^	102.4 (7)
C7—C8—H8	119.5	H25C—C25A—H25D	109.2
C7—C8—C9	121.1 (3)	C26A^iv^—C25A—H25C	111.3
C9—C8—H8	119.5	C26A^iv^—C25A—H25D	111.3
C4—C9—C10	122.4 (2)	N4—C26—H26C	107.0
C8—C9—C4	119.7 (2)	N4—C26—H26D	107.0
C8—C9—C10	117.9 (2)	C25^iv^—C26—N4	121.2 (11)
O3—C10—C9	115.8 (2)	C25^iv^—C26—H26C	107.0
O4—C10—O3	122.2 (2)	C25^iv^—C26—H26D	107.0
O4—C10—C9	122.0 (2)	H26C—C26—H26D	106.8
N1—C11—H11	118.6	N4A—C26A—H26A	105 (5)
N1—C11—C12	122.7 (3)	N4A—C26A—H26B	111 (5)
C12—C11—H11	118.6	C25A^iv^—C26A—H26A	94 (5)
C11—C12—H12	120.4	C25A^iv^—C26A—H26B	114 (5)
C13—C12—C11	119.1 (3)	H26A—C26A—H26B	125 (7)
C13—C12—H12	120.4	H1WA—O1W—H1WB	104.5
C12—C13—C14	118.4 (3)	H2WA—O2W—H2WB	109.4
C12—C13—C16	121.7 (3)	H3WA—O3W—H3WB	104.5
C14—C13—C16	119.7 (3)	H4WA—O4W—H4WB	104.5
C13—C14—H14	120.6		
			
Zn1—O1—C1—O2	−95.1 (5)	C14—C13—C16—N2	116.2 (3)
Zn1—O1—C1—C2	85.7 (6)	C15—N1—C11—C12	−0.2 (4)
Zn1^i^—O2—C1—O1	27.7 (4)	C16—N2—C17—C18^iii^	−132.7 (3)
Zn1^i^—O2—C1—C2	−153.07 (19)	C16—N2—C18—C17^iii^	132.5 (3)
Zn1^v^—O3—C10—O4	−10.6 (3)	C16—C13—C14—C15	174.1 (3)
Zn1^v^—O3—C10—C9	169.00 (18)	C17—N2—C16—O5	−176.5 (3)
Zn1—N1—C11—C12	−173.8 (2)	C17—N2—C16—C13	2.5 (5)
Zn1—N1—C15—C14	174.3 (2)	C17—N2—C18—C17^iii^	−54.2 (4)
Zn1—N3—C19—C20	−172.3 (4)	C18—N2—C16—O5	−4.0 (5)
Zn1—N3—C23—C22	173.9 (3)	C18—N2—C16—C13	175.0 (3)
O1—C1—C2—C3	−179.8 (3)	C18—N2—C17—C18^iii^	54.4 (4)
O2—C1—C2—C3	0.9 (4)	C19—N3—C23—C22	2.3 (5)
N1—C11—C12—C13	−0.6 (4)	C19—C20—C21—C22	3.5 (6)
N3—C19—C20—C21	−2.3 (7)	C19—C20—C21—C24	176.0 (4)
C1—C2—C3—C4	175.9 (3)	C20—C21—C22—C23	−2.0 (6)
C2—C3—C4—C5	30.6 (4)	C20—C21—C24—O6	−75.0 (5)
C2—C3—C4—C9	−148.3 (3)	C20—C21—C24—N4	90.5 (6)
C3—C4—C5—C6	−174.9 (3)	C20—C21—C24—N4A	120.4 (6)
C3—C4—C9—C8	174.1 (2)	C21—C22—C23—N3	−0.9 (6)
C3—C4—C9—C10	−5.3 (4)	C22—C21—C24—O6	97.2 (5)
C4—C5—C6—C7	0.3 (5)	C22—C21—C24—N4	−97.4 (6)
C4—C9—C10—O3	−142.4 (3)	C22—C21—C24—N4A	−67.5 (7)
C4—C9—C10—O4	37.1 (4)	C23—N3—C19—C20	−0.7 (6)
C5—C4—C9—C8	−4.9 (4)	C24—N4—C25—C26^iv^	168.1 (12)
C5—C4—C9—C10	175.8 (3)	C24—N4—C26—C25^iv^	−168.1 (14)
C5—C6—C7—C8	−3.9 (5)	C24—N4A—C25A—C26A^iv^	−100.9 (10)
C6—C7—C8—C9	3.0 (4)	C24—N4A—C26A—C25A^iv^	100.1 (9)
C7—C8—C9—C4	1.5 (4)	C24—C21—C22—C23	−174.3 (3)
C7—C8—C9—C10	−179.2 (2)	C25—N4—C24—O6	−14.5 (10)
C8—C9—C10—O3	38.2 (3)	C25—N4—C24—C21	−179.6 (6)
C8—C9—C10—O4	−142.2 (3)	C25—N4—C26—C25^iv^	29.8 (17)
C9—C4—C5—C6	4.0 (4)	C25A—N4A—C24—O6	−175.9 (7)
C11—N1—C15—C14	0.8 (4)	C25A—N4A—C24—C21	−11.5 (11)
C11—C12—C13—C14	0.8 (4)	C25A—N4A—C26A—C25A^iv^	−70.2 (9)
C11—C12—C13—C16	−173.5 (3)	C26—N4—C24—O6	−176.1 (7)
C12—C13—C14—C15	−0.2 (4)	C26—N4—C24—C21	18.8 (11)
C12—C13—C16—O5	109.4 (3)	C26—N4—C25—C26^iv^	−28.5 (16)
C12—C13—C16—N2	−69.6 (4)	C26A—N4A—C24—O6	15.2 (11)
C13—C14—C15—N1	−0.6 (4)	C26A—N4A—C24—C21	179.7 (7)
C14—C13—C16—O5	−64.7 (4)	C26A—N4A—C25A—C26A^iv^	69.0 (10)

Symmetry codes: (i) −*x*, −*y*+1, −*z*+1; (ii) *x*−1, *y*, *z*; (iii) −*x*−1, −*y*+1, −*z*+2; (iv) −*x*, −*y*+2, −*z*; (v) *x*+1, *y*, *z*.

### Hydrogen-bond geometry (Å, º)

**Table:**

*D*—H···*A*	*D*—H	H···*A*	*D*···*A*	*D*—H···*A*
O2*W*—H2*WA*···O3^ii^	0.87	1.87	2.732 (4)	172
O2*W*—H2*WB*···O3*W*	0.87	1.92	2.770 (7)	165
O3*W*—H3*WA*···O4*W*	0.87	1.85	2.585 (13)	140
O3*W*—H3*WB*···O5^vi^	0.87	2.08	2.888 (5)	154

Symmetry codes: (ii) *x*−1, *y*, *z*; (vi) −*x*, −*y*+1, −*z*+2.
